# Adult-type granulosa cell tumor of the testis: report of a case and review of literature

**DOI:** 10.11604/pamj.2017.26.198.11523

**Published:** 2017-04-04

**Authors:** Meriem Elbachiri, Amina Taleb, Nora Derrabi, Zineb Bouchbika, Nadia Benchakroun, Hassan Jouhadi, Nezha Tawfiq, Souha Sahraoui, Abdellatif Benider

**Affiliations:** 1 Mohamed VI Center of Cancer Treatment, Morocco; 2 Service d’Anatomie Pathologique, Chu Ibnou Rochd, Casablanca, Morocco

**Keywords:** Granulosa, tumour, testis, Adult-type granulosa cell tumour

## Abstract

Granulosa cell tumors is classified into juvenile and adult types and comprise less than 5% of ovarian tumors in women and are much rarer in men which only 45 have been previously reported. We report here a 40-year young man with a left testicular adult type granulosa cell tumor. The tumor measured 5.5X5X4cm; Immunohistochemical stains showed the tumor diffusely positive for inhibin and vimentin. Post operative CT scans shows a lomboaortic lymphnodes treated by four cycles of chemotherapy type BEP (bleomycin, etoposide, cisplatin). The thoraco abdominal CT scans post chemotherapy shows the disappearance of the right testicular nodule and the lomboaortic lymphnodes. 2 years after treatment, the patient is alive and well with no signs of recurrence. Our report highlights one more case of this very rare tumor of the testis, which is quite problematic In terms of prognosis and management, and for this reason seems to have attracted the interest of many researchers recently.

## Introduction

Granulosa cell tumor (GST) belongs to the sex-cord/stromal tumors of the gonads [1]. Two forms of GST have been recognized, namely, the typical adult type, and its variation, and the juvenile type [1]. Adult testicular granulosa cell tumors are extremely rare account for 1.6-6% of adult testicular tumors and occur somewhat more frequently in children and the clinical behavior of adult type granulosa cell tumors is difficult to predict [2]. Presently, these tumors appear to be slow-growing neoplasm with the potential to metastasize to distant sites years after initial diagnosis. Men who have undergone orchidectomy require extended follow-up because of the delayed metastatic potential of these tumors. Follow-up can be somewhat difficult, however, because there are no evidence-based guidelines available to help clinicians monitor their patients for metastatic disease [3]. Factors predictive of malignancy have yet to be well defined due to the very limited number of cases. We report an original case of adult type granulosa cell tumor in the testis reported for the first time at Mohamed VI center for cancer treatment in Casablanca for the first time and briefly review the previously published literature so as to improve the quality of management of this affection.

## Patient and observation

**Figure 1:**
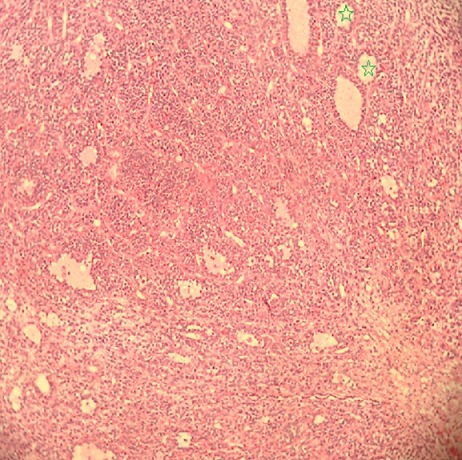
Low power view of granulose cell tumor shows microfollicular pattern of growth

**Figure 2:**
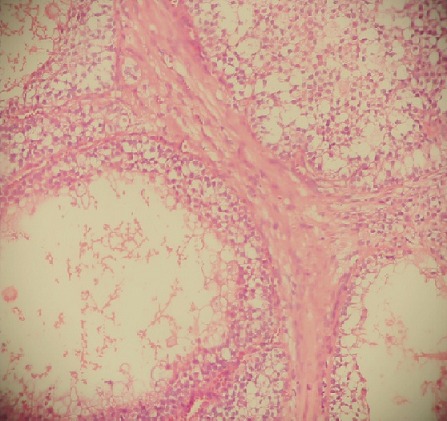
Granulosa cell tumor with a solid pattern of growth with call exners bodies (stars)

**Figure 3:**
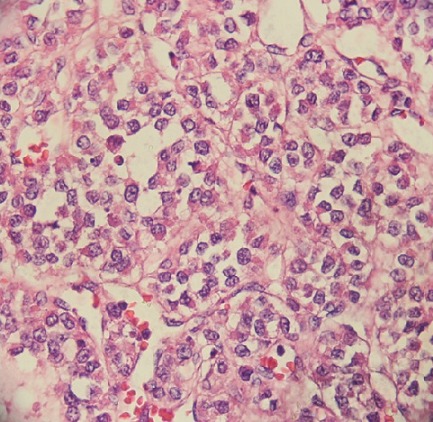
Granulosa cell tumor composed of tumor cells round to ovoid with irregular nuclear membranes and occasional nuclear

A 40-year-young man referred to the Mohamed VI center for cancer treatment in October 2014 with a 1-year history of a painless growing mass in the left testis without past history of trauma, infection, or lower urinary tract symptoms. Upon exploration, he reported a history of goiter, diabetes and scleroderma under treatment. Physical exams revealed a normal right epididymis and testis, and a 7 cm mass and hydrocele in the left testis, without hernia. Ultrasonography showed a 8x 10x7cm mass with solid and cystic components and a large hydrocele. Serum levels of alpha-fetoprotein, beta-hCG, and LDH were within normal ranges. He underwent inguinal surgical exploration of the left testis in June 2013, when a large testicular mass was found. A radical orchidectomy was performed, and the hydrocele fluid was analyzed. No frozen section biopsy was performed. Histopathological examination showed a 5.5X5X4cm yellow tumor. Final analysis revealed a granulosa cell tumor with oval nuclei having a longitudinal nuclear groove (Figure 1, Figure 2, Figure 3), focal invasion of the tunica albuginea and rete testis, and no invasion of surgical margins, spermatic cord, or epididymis. Cytological analysis of the hydrocele fluid was negative for tumor cells. Immunohistochemical tests were positive for inhibine and vimentin, but negative for epithelial membrane antigen. These findings strongly suggest the diagnosis of a granulosa cell tumor. His post operative blood tests were within normal limits, and the post operative abdominal and thoracic CT scans shows a right testis swollen with presence of nodules on the path of the left spermatic cord associated with a lomboaortic lymphnodes (Figure 1, Figure 2, Figure 3).the patient underwent 4 cycles of chemotherapy type BEP (bleomycin, etoposide,cisplatin).The abdominal and thoracic CT scans post chemotherapy shows the disappearance of the right testicular nodule and the lomboaortic lymphnodes. No signs of recurrence or metastasis have been identified after 6 months of follow-up.

## Discussion

Described for the first time in 1952, granulosa cell tumors are derived from epithelial elements of the sex cord, and they can be divided in juvenile or adult types [4]. Granulosa cell tumors affect mainly white males, usually as a painless testicular mass [5, 6] Gynecomastia is present in 25% of cases, due to hormonal abnormalities such as estrogen hypersecretion, or chromosomal abnormalities [4, 7]. Granulosa cell tumors affect both the ovaries and the testis, Only 29 cases of testicular adult type granulosa cell tumor have been previously reported [3, 6]. The diagnosis of sex cord-stromal tumors is mostly based on microscopic, morphologic features [5]. Morphological diagnosis is based primarily on the typical morphology of the granulosa cells with their coffeebean like, angulated and grooved nuclei. Macrofollicles ought to be present, the presence of the Call Exner bodies makes correct diagnosis easier; however, they are not always found and thus, are not indispensable to diagnosis [8]. The very rare fibrothecoma of the testis can be also mistaken for undifferentiated sex cord tumor since it manifests the same immunoreactivity as all other stromal tumors. This tumor, however, is composed of highly monotone spindle cells which are embedded in an acellular fibrous stroma [9] Immunohistochemically granulosa cell tumor is positive for inhibin, vimentin and calretinin,negative for epithelial membrane antigen (EMA), placental alkaline phosphotase,synaptophysin and lymphoid markers. Yolk sac tumor (YST) of mixed malignant germ cell tumors can show multiple growth patterns. However, YST is usually positive for PLAP,cytokeratin and AFP, albeit it can also be positive for inhibin [10]. The morphological features associated with malignant behaviors have not been well defined. Hanson et al.[11] reviewed all the reported cases of adult testicular granulosa cell tumors and found that only size greater than 5.0 cm reached statistical significance in association with malignancy. In the present case, even though the tumor was 7 centimeters in length, we did not find other malignant features, and the clinical behavior has been benign so far. Mitotic count and tumor necrosis did not reach statistical significance in predicting the tumor clinical behavior. More cases may be needed to refine the morphological features that may predict for the clinical behavior of the tumor [12]. Sites of metastases in male cases include retroperitoneal lymph nodes (most common), liver, bones, and the lungs [13]. Initial management is orchidectomy. Retroperitoneal lymphadenectomy has been additionally performed in a few cases where metastatic disease was suspected [13]. Metastatic disease may be managed with chemotherapy (etoposide alone or in combination with other agents) and adjuvant radiotherapy.

## Conclusion

Our report highlights one more case of this very rare tumor of the testis, which is quite problematic In terms of prognosis and management, and for this reason seems to have attracted the interest of many researchers recently. It’s necessary to identify prognostic factors that can reliably predict tumor behavior and to optimize methods of diagnosis and treatment .Long-term follow-up with a sufficient number of cases is recommended to define optimal treatment options since recurrence of the disease may appear late in the clinical course.

## References

[1] Majdoul soufya (2016). Récidive après dix ans de tumeur de la granulosa de l’ovaire: à propos de deux cas et revue de la littérature. The Pan African Medical Journal..

[2] Sun HD, Lin H, Jao MS, Wang KL, Liou WS, Hung YC (2012). A long-term follow-up study of 176 cases with adult-type ovarian granulosa cell tumors. Gynecol Oncol..

[3] Yi-Chan Chen L, Chang, Soong R (2012). A late recurring and easily forgotten tumor: ovarian granulosa cell tumor. World Journal of Surgical Oncology..

[4] Cheville JC (1999). Classification and pathology of testicular germ cell and sex cord-stromal tumors. Urol Clin North Am..

[5] Lazrak (2014). Granulosa-cell tumor of the ovary. International Journal of Innovation and Applied Studies.

[6] Al-Bozom IA, El-Faqih SR, Hassan SH (2000). Granulosa cell tumor of the adult type: a case report and review of the literature of a very rare testicular tumor. Arch Pathol Lab Med..

[7] Hisano M (2006). Granulosa cell tumor of the adult testis. Report case and review of the literature .CLINICS..

[8] Ortega Rojo A (2012). Recurrencia tardía del tumor de células de la granulosa: presentación de un caso. Gaceta Médica de México..

[9] Sourial MW, Sabbagh R (2013). A 17 year old male with a testicular fibrothecoma: a case report. Diagn Pathol.

[10] Cobellis L, Cataldi P, Reis FM (2001). Gonadal malignant germ cell tumors express immunoreactive inhibin/activin subunits. Eur J Endocrinol..

[11] Hanson JA, Ambaye AB (2011). Adult testicular granulosa cell tumor: a review of the literature for clinicopathologic predictors of malignancy. Arch Pathol Lab Med..

[12] Hisano M (2006). Granulosa cell tumor of the adult testis. Report of a case and review of the literature. CLINICS..

[13] Sekkate S, Kairouani M, Serji B, Errihani H (2014). Granulosa cell tumors of the ovary. Bulletin de Cancer..

